# Associations of BIA-Estimated Body Composition, Handgrip Strength, and Event-Defined Postural Control with Short-Range Police Precision-Shooting Accuracy: A Cross-Sectional Study

**DOI:** 10.3390/jfmk11030272

**Published:** 2026-07-15

**Authors:** Ana Campião, Pedro Aleixo, André Oliveira Massuça, João M. S. C. Abrantes, Luís Miguel Massuça

**Affiliations:** 1 Higher Institute of Police Sciences and Internal Security, 1300-663 Lisbon, Portugal; asrcampiao@psp.pt; 2 CIDEFES, Universidade Lusófona, and CIFI2D, Universidade do Porto, 1749-024 Lisbon and 4020-450 Porto, Portugal; pedro.aleixo@ulusofona.pt; 3 Bioengineering & Sustainability Research Group (BioRG), Faculty of Engineering, Lusófona University, 1749-024 Lisbon, Portugal; aomassuca@gmail.com; 4 School of Health Sciences of Alcoitão, 2649-506 Alcabideche, Portugal; joao.abrantes@scml.pt; 5 ICPOL—Police Research Center, Higher Institute of Police Science and Internal Security, 1300-663 Lisbon, Portugal

**Keywords:** body composition, centre of pressure, handgrip strength, marksmanship, police cadets, precision shooting, postural control

## Abstract

**Objectives:** This cross-sectional study examined associations between field bioelectrical impedance analysis (BIA)-estimated body composition, handgrip strength, event-defined centre-of-pressure (CoP) variables, and short-range police precision-shooting accuracy. **Methods**: Fifty-seven Police Officer Training Course cadets completed body-composition assessment, handgrip testing, and five-shot Glock 19 pistol tasks at 5 and 7 m. CoP variables were extracted during an event-defined aiming phase and a fixed 1 s post-discharge phase. **Results**: Shooting accuracy was higher at 5 m than at 7 m (*p* < 0.001). Higher BIA-estimated fat mass percentage was associated with lower shooting accuracy at both distances (*p* < 0.050), whereas handgrip strength was not associated with accuracy. Compared with the aiming phase, the post-discharge phase was characterized by greater CoP velocity-related measures and lower CoP amplitude and displacement (*p* < 0.010); these phase comparisons should be interpreted with caution because of differences in phase duration. Associations between CoP variables and shooting accuracy were generally weak. Sex-adjusted exploratory fixed-entry regression models showed modest explanatory capacity after internal validation (5 m: adjusted *R*^2^ = 0.281, LOOCV_*R*^2^ = 0.240; 7 m: adjusted *R*^2^ = 0.193, LOOCV_*R*^2^ = 0.131). BIA-estimated fat mass percentage and AP peak velocity during aiming were retained across distances, but these model-based findings should be interpreted as exploratory candidate associations rather than stable predictors. **Conclusions**: These findings suggest that field BIA-estimated body composition and task-specific postural regulation may be related to police precision-shooting accuracy but should be interpreted as correlates rather than determinants of performance.

## 1. Introduction

Police use of firearms represents one of the most demanding and sensitive components of law enforcement activity. Unlike sport shooting, which is typically performed under stable and predictable conditions, police shooting frequently occurs in operational contexts characterised by time pressure, uncertainty, and high cognitive and emotional demands. In these situations, shooting performance depends not only on technical proficiency but also on the ability to maintain motor control and postural regulation under stressful conditions [1,2]. Therefore, police firearms training aims to develop the technical foundations required for safe and effective weapon handling and accurate shot placement. In this context, precision-shooting tasks performed under controlled conditions are commonly used to assess marksmanship performance and its potential determinants in police cadets. However, shooting performance is recognised as a multifactorial phenomenon influenced by biomechanical, neuromuscular, physiological, and psychological factors [3]. Among these, postural stability has received particular attention in shooting-related research [3,4,5].

Previous studies in sport shooting have shown that reduced body sway and improved postural control are generally associated with greater shooting accuracy [3,4]. During the aiming phase, the ability to minimise the sway of the shooter–weapon system may contribute to more stable alignment and improved shot precision. Consequently, centre of pressure (CoP) measures have frequently been used to quantify postural stability in shooting tasks, particularly by analysing CoP displacement, velocity, and sway variability in the mediolateral (ML) and anteroposterior (AP) directions.

In addition to postural control, morphological characteristics and physical conditioning may also influence shooting performance. Body composition, particularly increased body fat, has been associated with poorer motor performance and reduced postural efficiency [5], whereas muscular strength, including handgrip strength, may contribute to weapon stabilisation and trigger control [4]. Therefore, handgrip strength may represent a practical and widely used field-based measure of upper-limb force capacity, given its direct involvement in firearm handling and its previously reported associations with shooting performance. Nevertheless, the magnitude and consistency of these associations remain unclear, and findings from sport shooting cannot be directly generalised to police contexts.

Research examining factors associated with precision-shooting performance in police cadets remains limited, particularly under controlled training conditions and at short engagement distances commonly used in firearms training programmes. Most existing evidence derives from sport-shooting populations [5], which differ substantially from police officers in task demands, environmental constraints, and training objectives. Moreover, it remains unclear whether associations reported between body composition, muscular strength, postural control, and shooting accuracy in sport shooters are also observable in police cadets. Specifically, few studies have simultaneously examined body composition, handgrip strength, and event-defined CoP variables as potential correlates of police precision-shooting performance. This represents an important knowledge gap, as identifying factors associated with shooting accuracy during training may contribute to a better understanding of performance variability in police cadets.

Therefore, the present study aimed to examine associations between BIA-estimated body composition, handgrip strength, event-defined CoP variables, and short-range police precision-shooting accuracy at 5 and 7 m in police cadets under controlled laboratory conditions. CoP variables were analysed during an aiming phase, defined from visible alignment and stabilisation until the frame immediately preceding shot discharge, and during a fixed 1 s post-discharge phase starting at shot discharge. Exploratory multivariable models of shooting accuracy were also examined at each distance. It was hypothesized that (i) lower body fat percentage, (ii) greater handgrip strength, and (iii) lower CoP displacement and velocity during the aiming phase would be associated with higher shooting accuracy. Given the limited evidence available in police populations, no specific hypotheses were formulated regarding the post-discharge CoP variables or the multivariable models.

## 2. Materials and Methods

### 2.1. Study Design

This study adopted a cross-sectional observational design to examine the relationships among BIA-estimated body composition, handgrip strength, event-defined CoP variables, and short-range police precision-shooting accuracy.

### 2.2. Participants

Fifty-seven police students enrolled in the CFOP at the Higher Institute of Police Sciences and Internal Security (ISCPSI) participated in the study (age, 27.67 ± 5.11 years; stature, 1.74 ± 0.06 m; body mass, 77.81 ± 11.89 kg). The sample comprised 43 men (age, 27.47 ± 4.95 years; stature, 176.03 ± 5.19 cm; body mass, 81.67 ± 10.26 kg) and 14 women (age, 28.29 ± 5.72 years; stature, 166.46 ± 4.32 cm; body mass, 65.96 ± 8.24 kg), and 56 were right-hand dominant. Participants had completed firearms training sessions and self-reported at least four years of shooting experience.

Participants were recruited by convenience sampling from different academic years of the CFOP. This choice stemmed from the applied nature of the research and the constraints inherent to the CFOP’s training context at ISCPSI. Even so, including all eligible participants maximised the internal representativeness of the analysed population. Accordingly, participants included all cadets who were in good health and available during the data collection periods announced by the research team; conversely, individuals were excluded if they had underlying health conditions or scheduling conflicts that prevented them from attending the designated collection sessions. No formal a priori sample-size calculation was performed. The sample size was determined by the finite number of eligible CFOP cadets who were available during the data-collection period. All eligible and available cadets who met the inclusion criteria and provided written informed consent were included.

Before data collection, all participants received detailed information about the study’s aims and procedures and provided written informed consent. Participation was voluntary, and anonymity and confidentiality were ensured throughout the study. The research protocol was approved by the Teaching Department and Scientific Council of ISCPSI (protocol code SECDE202500002ASP, number 173/SECDE/2025, 30 October 2025). All procedures complied with the Declaration of Helsinki.

### 2.3. Morphological and BIA-Estimated Body-Composition Assessment

Morphological and body-composition variables were assessed using anthropometric measurements and bioelectrical impedance analysis (BIA). BIA-estimated body composition was assessed under field conditions. Participants were barefoot and wore the same operational police uniform condition during testing. Body mass used for BIA and BMI calculation included the uniform mass. Pre-assessment hydration, fasting status, caffeine/alcohol intake, and recent exercise were not standardised. Accordingly, fat mass percentage and muscle mass are reported as BIA-derived estimates rather than laboratory-standardised body-composition measures.

Body height (m) was measured using a portable stadiometer (seca 213, seca GmbH & Co. KG, Hamburg, Germany), with participants standing upright in the Frankfurt plane [6]. Body mass (kg), body mass index (BMI; kg/m^2^), BIA-estimated fat mass (%), and BIA-estimated muscle mass (kg) were obtained using a dual-frequency bioelectrical impedance analyser (Tanita InnerScan V BC-601 Gold, Tanita Ltd., Amsterdam, The Netherlands). The analyser was used in standard mode, and age, sex, and stature were entered according to the manufacturer’s instructions before each assessment.

### 2.4. Handgrip Strength Assessment

**Figure 1:**
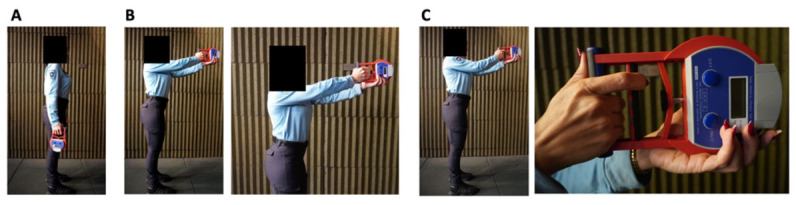
Representative assessment positions for handgrip and finger-grip strength: handgrip strength with the upper limb alongside the body (**A**), dominant-hand handgrip strength with the shoulder flexed at 90° and the elbow extended (**B**), and dominant-index-finger strength in the shooting position (**C**).

Handgrip strength was assessed using a digital hand dynamometer (T.K.K.5401, Takei, Tokyo, Japan). Three testing conditions were evaluated: (i) dominant and non-dominant handgrip strength with the arm extended alongside the body (Figure 1A); (ii) dominant handgrip strength with the shoulder flexed at 90° and the elbow extended (Figure 1B); and (iii) index-finger strength in the shooting position (Figure 1C). Participants were instructed to exert maximal force for 5 s in each trial, i.e., the duration reported in the literature for assessing handgrip strength [7]. Two attempts were performed for each condition, separated by approximately 60 s of rest. The same rest interval was provided before changing to the next handgrip condition. The mean value of the two attempts was used for analysis.

### 2.5. Shooting Accuracy Assessment

**Figure 2:**
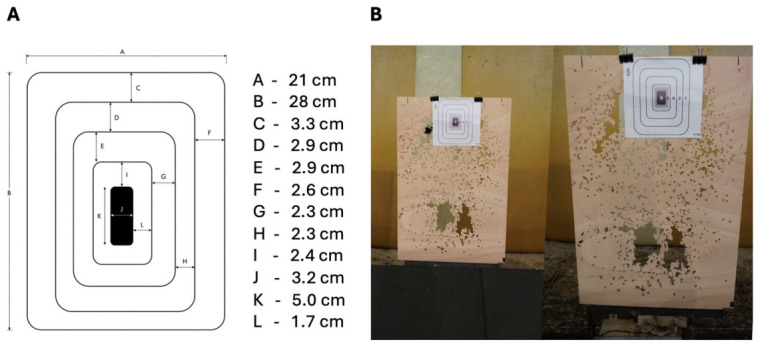
Target used in precision shooting assessment, with indication of scoring zones and their respective dimensions (**A**), and target layout on the shooting range (**B**).

Precision shooting performance was evaluated using a Glock 19 pistol (9 × 19 mm calibre; GLOCK Ges.m.b., Deutsch-Wagram, Austria) at distances of 5 and 7 m, and the order of the 5 and 7 m conditions was randomised using block randomisation, with 26 participants starting at 5 m and 31 at 7 m. Participants were assessed barefoot while wearing the operational police uniform (USO2) and fired five shots at each distance, yielding a maximum score of 25 points per condition, i.e., shooting accuracy for each shot was scored from 0 to 5 points based on the target zone hit (Figure 2A). Participants performed the shooting task barefoot to allow more accurate acquisition of CoP data from the pressure platform.

Target scores were determined by a trained firearms instructor using the official zone-based scoring system. When a shot touched the boundary between two scoring zones, the higher score was assigned. The scorer was blinded to participants’ BIA-estimated body composition, handgrip, and CoP data.

Targets were positioned with their centres at 1.43 m above the ground (Figure 2B), in accordance with the police training programme. Participants initiated the procedure upon verbal command, i.e., (i) at the command “ammunition”, participants inserted five rounds into the magazine and then inserted it into the weapon; (ii) at the command “load”, they pulled the slide back, then remained in a resting position with the weapon pointed forward, close to the abdominal region, and with their elbows bent; and (iii) at the command “fire”, they adopted the firing position, acquired the aiming point, and fired. Participants had 10 s to complete each shot after the verbal command “fire”; after each shot, they returned to the resting position, repeating the procedure until the five shots planned for each distance were completed. Once the corresponding series was finished, the equipment was adjusted for the next distance, and the same protocol was repeated.

### 2.6. Postural Control During Shooting

Postural control during shooting was assessed in the ISCPSI indoor shooting range using a Footscan^®^ pressure platform (RSscan International, Olen, Belgium; 50 Hz sampling frequency; 1068 mm × 418 mm × 12 mm). This equipment has been used in several studies for collecting CoP data [8,9].

In this study, participants adopted a barefoot standing shooting position, predominantly a modern isosceles stance [10], while performing the shooting tasks.

CoP data were recorded throughout the shooting sequence, and each trial was simultaneously filmed using a Canon EOS 550D camera (Canon Inc., Tokyo, Japan) at 25 Hz. A manual marker applied to the pressure-platform recording at the verbal command “fire” provided a temporal reference for synchronising the video and CoP data. Because video and CoP data were acquired at 25 and 50 Hz, respectively, event timing was determined at the frame level.

The AP axis was defined as the direction parallel to the line of fire, corresponding to forward–backward displacement relative to the target, whereas the ML axis was defined as the left–right displacement perpendicular to the line of fire.

Shot discharge was identified from the audio signal and/or the video frame showing visible weapon recoil and was used as the temporal boundary between the aiming and post-discharge phases. Because no trigger-force sensor was used, trigger execution could not be isolated from the final part of the aiming phase. Accordingly, any trigger-related movement occurring before shot discharge was included within the aiming phase rather than analysed as a separate phase.

Phase segmentation was performed by one trained rater using frame-by-frame visual inspection in Filmora (version 15) [11]. The aiming phase began in the first frame in which the participant aligned the body segments and pistol with the target and adopted a visibly stable shooting position, without gross body or weapon repositioning. The aiming phase ended at the frame immediately preceding shot discharge. The post-discharge phase was defined as a fixed 1 s window starting at shot discharge. Thus, the post-discharge phase captured shot discharge and the immediate post-discharge postural response, but did not include pre-discharge trigger actuation as a separate analysed interval. The use of one trained rater was intended to ensure procedural consistency across all trials. However, no intra- or inter-rater reliability analysis was performed, because the available analytical dataset contained only the final segmented and averaged phase-specific CoP variables and did not include repeated frame-level segmentations. Therefore, segmentation repeatability could not be quantified retrospectively.

Because the aiming phase was event-defined and had variable duration, whereas the post-discharge phase was a fixed 1 s window, phase comparisons involving duration-sensitive variables, particularly total displacement, were interpreted with caution. Mean velocity and velocity/amplitude ratios were considered less directly dependent on window duration and were therefore emphasised when interpreting phase-related differences.

CoP data were computed using the Biomechanical Stability Program [12], yielding the following variables: (i) CoP AP and ML amplitudes (mm; difference between the maximum and minimum values of the trajectory on the AP and ML axis) [13]; (ii) CoP AP and ML mean velocities (mm/s; average of the AP and ML velocity values, determined using the finite-difference method from CoP positions) [13]; (iii) CoP AP and ML peak velocities (mm/s; highest AP and ML velocity value) [13]; (iv) total displacement (mm; total CoP path length within the analysed phase, obtained by summing successive point-to-point displacements) [14]; (v) ellipse area (mm^2^; area of the 95% prediction ellipse, representing the overall trajectory dispersion) [13,15]; (vi) axis a (mm; major axis of the ellipse, corresponding to the direction of greatest dispersion of the trajectory) [15,16]; (vii) axis b (mm; minor axis of the ellipse, corresponding to the direction of least dispersion of the trajectory) [15,16]; (viii) AP and ML root mean square (RMS; mm) [13]; (ix) ML/AP amplitude (ratio between CoP ML amplitude and the CoP AP amplitude, allowing for identification of the predominant displacement plane; values > 1 indicate a predominance of ML displacement, while values < 1 indicate AP predominance); (x) ML/AP velocity (ratio between CoP ML mean velocity and the CoP AP mean velocity; values > 1 indicate a predominance of dynamic activity in the ML axis, while values < 1 indicate AP predominance); (xi) AP velocity/amplitude (ratio between the CoP AP mean velocity and the CoP AP amplitude; values > 1 indicate greater corrective dynamics in relation to the magnitude of the displacement on the AP axis, while values < 1 indicate displacement of greater amplitude relative to the dynamic activity of the CoP on that axis); (xii) ML velocity/amplitude (ratio between the CoP ML mean velocity and the CoP ML amplitude; values > 1 indicate greater corrective dynamics in relation to the magnitude of the displacement on the ML axis, whereas values < 1 indicate displacement of greater amplitude relative to the dynamic activity of the CoP on that axis); (xiii) AP and ML variances (mm^2^; variance of the CoP AP position, quantifying the mean quadratic dispersion around the mean position) [13]; (xiv) ML/AP covariance (covariance between the ML and AP positions, indicating the degree of association between ML and AP displacement) [15].

No additional filtering was applied beyond the manufacturer/software processing. Because peak-velocity variables are sensitive to sampling frequency, filtering, and finite-difference procedures, peak CoP velocity was treated as an exploratory descriptor and interpreted cautiously.

For each participant, distance, and phase, CoP variables were first computed for each shot and then averaged across the five shots before statistical analysis. The duration of the aiming phase was retained for each shot and averaged across participants and distances. The post-discharge phase was fixed at one second for all shots.

### 2.7. Statistical Analysis

Descriptive statistics are presented as mean ± standard deviation.

Distributional assumptions were assessed using visual inspection of histograms and Q–Q plots, together with Shapiro–Wilk tests. Because several shooting-accuracy and CoP-derived variables showed departures from normality, associations between physical, postural-control, and shooting-accuracy variables were analysed using Spearman’s rank correlation coefficients. For paired comparisons, the normality of paired-difference scores was inspected. When distributional deviations were observed, Wilcoxon signed-rank tests were used as sensitivity analyses to verify whether the interpretation of the paired comparisons was materially altered. Because the Wilcoxon sensitivity analyses did not materially alter the interpretation of the main paired-comparison findings, the paired-sample *t*-test results are reported for consistency with the effect-size estimates.

Paired-sample *t*-tests were used to evaluate whether: (i) shooting accuracy differed between 7 m and 5 m; (ii) CoP variables differed between the post-discharge and aiming phases at each shooting distance; and (iii) CoP variables differed between 7 m and 5 m within each phase. Effect sizes were calculated using Hedges’ *g* [17], with values of approximately 0.20, 0.50, and 0.80 interpreted as small, medium, and large effects, respectively.

Because no formal a priori sample-size calculation was performed, the analyses were interpreted as exploratory. As an interpretive sensitivity estimate, with *n* = 57, a two-tailed correlation analysis at α = 0.05 would have approximately 80% power to detect correlations of about |r| ≈ 0.36 or larger. Therefore, smaller associations, particularly weak CoP–accuracy relationships, may have gone undetected.

Associations between morphological and body-composition variables, handgrip strength, CoP variables, and shooting accuracy were analysed using Spearman’s correlation coefficients. In addition, partial correlations were computed to examine the associations between physical fitness attributes and precision shooting performance, controlling for sex as a covariate. Correlation coefficients were interpreted according to Cohen [18], with values of 0.10, 0.30, and 0.50 considered weak, moderate, and strong, respectively.

Because the study was exploratory and several bivariate tests were performed, unadjusted *p*-values are reported and interpreted together with effect sizes and 95% confidence intervals. As a sensitivity analysis, Benjamini–Hochberg false-discovery-rate (BH-FDR)-adjusted *p*-values were calculated within pre-specified families of tests: physical-attribute bivariate correlations, sex-adjusted physical-attribute partial correlations, phase-comparison tests at each distance, and distance-/phase-specific CoP–accuracy correlations. Findings with unadjusted *p* < 0.05 but *q* ≥ 0.05 were described as nominal and interpreted as hypothesis-generating rather than confirmatory evidence.

Exploratory multivariable linear regression models were fitted separately for 5 m and 7 m shooting accuracy. Given the limited sample size relative to the number of measured descriptors, the regression analyses were not intended to develop definitive predictive equations or to provide confirmatory evidence of independent determinants of shooting accuracy. Instead, they were treated as parsimonious, hypothesis-generating candidate models. Following the preliminary exploratory model-building stage, the final reported models were re-estimated using a fixed-entry approach. This avoided further automatic variable selection in the final reported models and allowed sex to be forced into each model as a covariate. For the 5 m model, sex, BIA-estimated fat mass percentage, AP peak CoP velocity during the aiming phase, ML velocity/amplitude during the post-discharge phase, and ML/AP amplitude during the post-discharge phase were entered simultaneously. For the 7 m model, sex, BIA-estimated fat mass percentage, and AP peak CoP velocity during the aiming phase were entered simultaneously. Sex was included because it may influence body composition, strength-related characteristics, and shooting performance. Sex was coded as 0 = female and 1 = male. Regression assumptions were inspected using residual plots, Q–Q plots, homoscedasticity checks, Cook’s distance, tolerance, and variance inflation factor values. To assess internal model performance and potential optimism, bootstrap resampling with 5000 samples and leave-one-out cross-validation were performed. Percentile bootstrap 95% confidence intervals were calculated for regression coefficients. Apparent *R*^2^, adjusted *R*^2^, LOOCV_*R*^2^, LOOCV_RMSE, and bootstrap optimism-corrected *R*^2^ were reported to evaluate model performance. Given the exploratory nature of the study, the regression models were interpreted as hypothesis-generating candidate associations rather than confirmatory predictive models.

Statistical analyses were performed using IBM SPSS Statistics 31.0, JASP 0.19.3 [19], and custom scripts for bootstrap and leave-one-out cross-validation procedures, with statistical significance set at *p* < 0.05.

## 3. Results

### 3.1. Performance in Precision Police Shooting at 5 and 7 m

Precision shooting performance was higher at 5 m than at 7 m (5 m, 18.11 ± 5.85 points; 7 m, 15.05 ± 6.64 points; *p* < 0.001), with an effect size approaching the conventional medium threshold (Table 1).

### 3.2. Morphological Body-Composition and Handgrip Variables Associated with Police Shooting at 5 and 7 m

BMI and BIA-estimated fat mass percentage showed nominal negative bivariate associations with shooting accuracy at both distances (Table 2). Body mass showed a weak negative association at 5 m (*r* = −0.261) that was at the conventional *p* = 0.050 threshold, and a nominal negative association at 7 m (*r* = −0.279, *p* = 0.036). After controlling for sex in partial correlations, body mass, BMI, and BIA-estimated fat mass percentage remained negatively associated with shooting accuracy at both distances, with BIA-estimated fat mass percentage showing the strongest sex-adjusted correlations (5 m: *r* = −0.407, *p* = 0.002; 7 m: *r* = −0.409, *p* = 0.002). No nominal associations were observed for BIA-estimated muscle mass or handgrip strength variables.

In the BH-FDR sensitivity analysis, none of the 22 unadjusted bivariate physical-attribute correlations remained significant after correction (lowest *q* = 0.183). In contrast, within the 22 sex-adjusted partial correlations, BIA-estimated fat mass percentage remained associated with shooting accuracy at both 5 m and 7 m (both *q* = 0.022), whereas body mass and BMI associations were nominal only after correction (*q* ≥ 0.051).

### 3.3. CoP Behaviour During Precision Police Shooting at 5 and 7 m

At both shooting distances, the event-defined aiming phase and the fixed 1 s post-discharge phase showed distinct CoP behaviour (Table 3). The post-discharge phase showed higher CoP mean velocity and velocity/amplitude ratios than the aiming phase. Conversely, lower CoP amplitude, total displacement, and several displacement-related parameters were observed during the post-discharge phase. Because the aiming phase was longer and event-defined, whereas the post-discharge phase was fixed at one second from shot discharge, differences in total displacement and other duration-sensitive variables should be interpreted with caution.

At 5 m, the post-discharge phase showed increases in ML and AP CoP mean velocity and in ML and AP velocity/amplitude ratios, together with decreases in ML and AP amplitude, ML peak velocity, total displacement, ML/AP amplitude, ML/AP velocity, and ML/AP covariance. At the unadjusted level, a similar pattern was observed at 7 m, with increases in ML and AP CoP mean velocity and velocity/amplitude ratios, and decreases in ML and AP amplitude, ML and AP peak velocity, total displacement, axis a, axis b, AP RMS, ML/AP amplitude, ML/AP velocity, and ML/AP covariance.

The mean duration of the aiming phase was 3.79 ± 1.29 s at 5 m (CV = 33.9%) and 3.77 ± 1.20 s at 7 m (CV = 31.7%). The post-discharge phase was fixed at one second at both distances. Because no repeated segmentation was available, these coefficients of variation should be interpreted as descriptive variability in the event-defined aiming duration rather than as segmentation reliability estimates. This variability likely reflects both true behavioural differences in the time required to align, stabilise, and discharge the shot and potential unquantified variability in manual event detection.

BH-FDR correction did not materially alter the main phase-comparison pattern. At 5 m, the phase differences that remained significant after correction were ML and AP amplitude, ML and AP mean velocity, ML peak velocity, total displacement, ML/AP amplitude, ML/AP velocity, ML velocity/amplitude, AP velocity/amplitude, and ML/AP covariance. At 7 m, the phase differences that remained significant after BH-FDR correction were ML and AP amplitude, ML and AP mean velocity, ML peak velocity, total displacement, axis a, axis b, AP RMS, ML/AP amplitude, ML/AP velocity, ML velocity/amplitude, AP velocity/amplitude, and ML/AP covariance. AP peak velocity showed a nominal decrease at the unadjusted level (*p* = 0.038), but this difference did not remain significant after BH-FDR correction (*q* = 0.056).

Complementary, it was also observed that no distance-related differences in CoP variables reached the conventional statistical threshold between 5 and 7 m in either the aiming or post-discharge phase. This conclusion was unchanged after Benjamini–Hochberg false-discovery-rate correction across the 38 distance-comparison tests, with all adjusted *q*-values equal to 0.960 (Appendix A; Table A1).

CoP–accuracy correlations were generally weak. Two nominal positive correlations were observed at 5 m for AP peak velocity during the aiming phase (*r* = 0.270, *p* = 0.042) and post-discharge phase (*r* = 0.269, *p* = 0.043). However, neither association survived BH-FDR correction within the corresponding 19-test distance-/phase-specific families (*q* = 0.350 and *q* = 0.552, respectively), nor when all CoP-accuracy tests were considered together. No CoP variable was associated with 7 m shooting accuracy after correction.

After Benjamini–Hochberg false-discovery-rate correction across the 76 CoP–accuracy correlations, no CoP variable remained associated with shooting accuracy at *q* < 0.05. Accordingly, the nominal AP peak-velocity associations observed at 5 m during aiming and post-discharge should be interpreted as exploratory.

Spearman correlations between CoP variables and police shooting accuracy at 5 and 7 m, considering the aiming and post-discharge phases, are presented in Table 4.

### 3.4. Sex-Adjusted Exploratory Regression Models

Sex-adjusted exploratory fixed-entry regression models were fitted for shooting accuracy at 5 and 7 m (Table 5). The 5 m model retained the same substantive predictors identified in the preliminary exploratory modelling stage, with sex entered as a forced covariate. The model was statistically significant, *F*(5,51) = 5.371, *p* < 0.001, and showed modest explanatory capacity (*R* = 0.587, *R*^2^ = 0.345, adjusted *R*^2^ = 0.281). Internal validation indicated reduced but still positive model performance, with LOOCV_*R*^2^ = 0.240 and LOOCV_RMSE = 5.052. Bootstrap optimism-corrected *R*^2^ was 0.237.

In the 5 m model, BIA-estimated fat mass percentage, AP peak velocity during aiming, and ML velocity/amplitude during the post-discharge phase showed bootstrap 95% confidence intervals that did not include zero. ML/AP amplitude during the post-discharge phase showed a negative model coefficient, but its bootstrap confidence interval crossed zero, indicating lower coefficient stability. Sex was not statistically significant at the conventional threshold.

The 7 m model included sex, BIA-estimated fat mass percentage, and AP peak velocity during aiming. The model was statistically significant, *F*(3,53) = 5.468, *p* = 0.002, with *R* = 0.486, *R*^2^ = 0.236, and adjusted *R*^2^ = 0.193. Internal validation yielded LOOCV_*R*^2^ = 0.131 and LOOCV_RMSE = 6.136, and bootstrap optimism-corrected *R*^2^ was 0.156.

In the 7 m model, BIA-estimated fat mass percentage and AP peak velocity during aiming showed bootstrap 95% confidence intervals that did not include zero, whereas sex did not. Across both distances, the inclusion of sex did not materially alter the direction of the associations involving BIA-estimated fat mass percentage or AP peak velocity during aiming. Nevertheless, the reduction in internally validated performance compared with apparent *R*^2^ supports interpreting these models as exploratory and hypothesis-generating rather than confirmatory predictive models.

## 4. Discussion

This study contributes to the limited literature examining factors associated with precision-shooting performance in police cadets under controlled training conditions. Understanding the physical and postural characteristics associated with marksmanship performance may help inform future research and training strategies in police firearms instruction. Therefore, the present study examined the associations among BIA-estimated body composition, handgrip strength, event-defined centre-of-pressure (CoP) variables, and short-range police precision-shooting accuracy among cadets attending a Police Officer Training Course. Overall, shooting accuracy was higher at 5 m than at 7 m, BIA-estimated fat mass percentage emerged as the most consistent correlate of performance, handgrip strength was not associated with accuracy, and CoP–accuracy relationships were generally weak.

The higher shooting accuracy observed at 5 m compared with 7 m is consistent with the perceptual and motor demands of precision shooting. Although both distances represent short-range police training scenarios, increasing target distance reduces tolerance for aiming error because small deviations in weapon alignment produce greater displacement of the shot’s impact on the target. Shooting accuracy, therefore, depends on the integration of visual alignment, trigger control, postural regulation, and task-specific motor coordination [1,3,21,22]. In police-related contexts, shooting performance is also influenced by task constraints, decision-related demands, and stress responses. However, the present protocol was conducted under controlled precision-shooting conditions rather than under operational stress [2,23].

One of the most consistent findings was a negative association between BIA-estimated body-composition indicators and shooting accuracy. Higher BMI and BIA-estimated fat mass percentage were nominally associated with lower shooting scores at both distances, and these associations remained evident after controlling for sex. After BH-FDR correction, however, only the sex-adjusted associations involving BIA-estimated fat mass percentage remained below the correction threshold. Therefore, the strongest inference from the present data concerns BIA-estimated fat mass percentage as a sex-adjusted correlate of shooting accuracy, rather than a broad effect of body size or body-composition indicators. Because body composition was estimated using BIA under field conditions, and without standardisation of hydration, fasting status, caffeine/alcohol intake, or recent exercise, these findings should be interpreted as associations involving field-estimated body-composition indicators rather than laboratory-standardised adiposity measures. In the multivariable analyses, BIA-estimated fat mass percentage showed a statistically significant negative coefficient in both the 5 m model (B = −0.291, *p* = 0.021; bootstrap 95% CI, −0.518 to −0.055) and the 7 m model (B = −0.416, *p* = 0.004; bootstrap 95% CI, −0.655 to −0.183), after accounting for the other variables included in each model. Nevertheless, BIA-estimated fat mass percentage should be interpreted as a relevant correlate of precision-shooting performance in police cadets under controlled training conditions rather than as an isolated determinant of performance.

Several mechanisms may explain the association between higher BIA-estimated fat mass percentage and lower shooting accuracy. Greater adiposity may alter body mass distribution, increase the postural demands required to stabilise the shooter–weapon system, and reduce movement economy during standing aiming tasks. It may also be associated with less efficient sensorimotor regulation, making it more difficult to maintain a stable firing posture and perform fine corrective adjustments during aiming. Evidence from shooting and aiming-based tasks indicates that body stability and biomechanical regulation may be relevant to performance, although the specific mechanisms are task-dependent [5,24,25]. Nevertheless, these mechanisms were not directly measured in the present study. Accordingly, the observed associations should be interpreted as indirect and hypothesis-generating, particularly because the cross-sectional design does not allow for causal inference.

In contrast, muscle mass and handgrip strength were not significantly associated with shooting accuracy. This suggests that, in this sample of trained police students, maximal force production was not a limiting factor for short-range precision shooting. Although handgrip and shoulder strength may be relevant to weapon handling, upper-limb stabilisation, and recoil management, maximal grip force may not directly translate into improved shooting accuracy in a controlled short-range task [4,26]. Shooting accuracy may depend more strongly on grip consistency, trigger control, sight alignment, upper-limb steadiness, breathing regulation, and fine sensorimotor coordination than on maximal handgrip strength. Excessive grip tension may even interfere with trigger sensitivity or increase tremor, although this was not directly assessed in the present study.

The aiming and post-discharge phases exhibited distinct CoP profiles. However, these findings should be interpreted cautiously because the aiming phase was event-defined and variable in duration, whereas the post-discharge phase consisted of a fixed 1 s interval beginning at shot discharge. Consequently, comparisons involving duration-sensitive variables may partly reflect differences in phase structure rather than differences in postural regulation per se. Therefore, the observed phase-specific CoP patterns are best viewed as descriptive characteristics of the analysed task phases, and not as evidence of superior or inferior postural stability or efficiency. Furthermore, because the post-discharge phase began at shot discharge, the corresponding CoP variables should be interpreted as reflecting immediate post-discharge responses rather than determinants of shot placement.

An additional methodological consideration concerns the reliability of phase segmentation. Although segmentation was performed frame-by-frame using predefined criteria by a trained rater, no quantitative intra- or inter-rater reliability estimate was available. Therefore, some portion of the variability in phase-specific CoP descriptors, particularly variables sensitive to temporal window definition, may reflect unquantified variability in manual event detection. This issue is especially relevant for the event-defined aiming phase, whose duration varied substantially between trials and participants.

Despite clear differences in CoP behaviour between phases, correlations between CoP variables and shooting accuracy were generally weak. The only nominal positive correlations were observed between 5 m shooting accuracy and AP peak velocity during the aiming and post-discharge phases, but these associations did not survive BH-FDR correction. Accordingly, they should be interpreted as exploratory signals rather than robust bivariate associations. These findings contrast with some sport-shooting studies that have reported relationships among lower body sway, rifle stability, aiming behaviour, and shooting performance [24,25,27]. However, the present study involved police students, short-range pistol shooting, a five-shot scoring protocol, and event-defined CoP phases, which limit direct comparison with sport-shooting research. Furthermore, shooting accuracy was based on only five shots at each distance, and the reliability of this score was not formally assessed. Consequently, measurement error in the outcome variable may have reduced the ability to detect true associations and attenuated the magnitude of the observed correlations. Therefore, the weak CoP–accuracy relationships observed in the present study may reflect not only the absence of strong underlying associations but also the limited reliability of the shooting-accuracy measure. In addition, precision-shooting performance in police cadets under controlled training conditions is likely influenced by variables not captured by CoP data, including visual search behaviour, gaze control, trigger execution, attentional focus, stress regulation, and previous training exposure [1,2,28].

The absence of significant distance-related differences in CoP variables between 5 and 7 m also deserves consideration. Although shooting accuracy was lower at 7 m, CoP behaviour did not differ significantly between distances within either phase. This suggests that the performance decrement at 7 m may have been driven more by increased demands on visual–motor precision and reduced tolerance for aiming error than by detectable changes in whole-body CoP behaviour. In other words, similar postural control patterns may yield different scoring outcomes as the target distance increases. This reinforces the idea that CoP variables provide only a partial representation of shooting performance and should be interpreted alongside weapon motion, gaze, trigger control, and shot location measures.

The sex-adjusted exploratory fixed-entry regression models provided a limited multivariable perspective on short-range police precision-shooting accuracy. BIA-estimated fat mass percentage was retained with a negative coefficient in both distance-specific models, reinforcing its role as the most consistent body-composition correlate of shooting performance in the present sample. This finding is also consistent with the sex-adjusted partial-correlation analyses, in which BIA-estimated fat mass percentage showed the strongest and most consistent associations with shooting accuracy.

AP peak velocity during the aiming phase was also retained in both regression models, with a positive coefficient. However, this finding should be interpreted cautiously. AP peak velocity did not remain significant after BH-FDR correction in the bivariate CoP–accuracy analyses, and greater peak CoP velocity during aiming would not typically be expected to directly facilitate accurate shot placement. Therefore, this association may reflect sample-specific model fitting, compensatory postural adjustments, or unmeasured aspects of trigger execution, weapon control, gaze behaviour, or shot timing.

The inclusion of sex as a forced covariate did not materially alter the direction of the model-based associations involving BIA-estimated fat mass percentage or AP peak velocity during aiming. Nevertheless, sex itself was not statistically significant in either model, and the small number of women limited more detailed sex-specific analyses. Internal validation further supported a cautious interpretation: LOOCV and bootstrap optimism-corrected estimates were lower than the apparent *R*^2^ values, indicating some degree of optimism, as expected in a small exploratory sample. Accordingly, these regression findings should be interpreted as internally evaluated exploratory candidate associations rather than stable or causal determinants of police shooting accuracy.

From a practical perspective, the findings suggest that firearms training and physical conditioning should be considered complementary components of police training programmes. Although causal relationships cannot be inferred, routine monitoring of BIA-estimated body composition may help identify trainees who could benefit from broader physical-conditioning strategies aimed at maintaining healthy body-composition profiles. Similarly, balance- and postural-control exercises could be incorporated into firearms-training programmes, particularly during static aiming drills, although their effectiveness for improving shooting accuracy remains to be established. Given the known influence of stress, gaze control, decision-making, and resilience on police performance, such approaches should ideally be integrated with scenario-based training that progressively incorporates cognitive, perceptual, and psychophysiological demands [1,2,29].

Several limitations should be acknowledged. No formal a priori sample-size calculation was performed because the study was conducted in a finite police academy training cohort and included all eligible and available cadets during the data-collection period. The sample size was therefore relatively small, and participants fired only five shots at each distance, which may have reduced the reliability and sensitivity of the shooting-accuracy measure. Consequently, the study may have been underpowered to detect small associations, particularly for CoP–accuracy relationships, and the regression findings should be interpreted as exploratory and hypothesis-generating.

In addition, several potentially influential factors were not standardised or quantified, including hydration status, recent fatigue, prior training load, psychological stress, sleep quality, and previous shooting experience. These factors may have contributed to inter-individual variability in both shooting performance and postural-control measures and therefore represent additional sources of unexplained variance.

The reliability of the target-scoring procedure was not formally assessed, and the five-shot score may have limited sensitivity for detecting subtle inter-individual differences in shooting performance.

The cross-sectional design precludes causal inference. The analyses were exploratory and involved multiple comparisons, although BH-FDR correction was used as a sensitivity analysis. The multivariable regression models were revised to include sex as a forced covariate and were internally evaluated using bootstrap resampling and leave-one-out cross-validation. Nevertheless, the sample size remained limited relative to the number of measured descriptors, and the regression models were based on a preliminary exploratory model-building stage. Therefore, the retained predictors may still be sample-dependent and should be interpreted as exploratory candidate correlates rather than stable predictive determinants of shooting accuracy.

Although sex was included in the revised regression models, the small number of women limited the possibility of sex-stratified analyses or sex-by-predictor interaction testing. Other potentially relevant covariates, including prior shooting experience, training volume, hand dominance, fatigue, stress, sleep quality, and psychological state, were not incorporated into the multivariable models and may have contributed to unexplained variance.

The shooting protocol was conducted barefoot on the pressure platform, which may limit its ecological validity compared with operational shooting with footwear. In addition, BIA-estimated body composition may be affected by hydration status, recent exercise, caffeine or alcohol intake, and other pre-assessment conditions that were not standardised. Although all participants were assessed under the same clothing condition, these non-standardised assessment conditions may have introduced measurement error and reduced the accuracy of the BIA-derived body-composition estimates. Consequently, the observed associations involving body-composition variables should be interpreted with caution and regarded as indicative rather than definitive evidence of a relationship between body composition and shooting accuracy.

Finally, phase segmentation was based on 25 Hz video analysis and was performed by a single trained rater using predefined operational criteria and frame-by-frame visual inspection. Although the use of one trained rater ensured procedural consistency across trials, no intra- or inter-rater reliability analysis was performed. Consequently, segmentation repeatability could not be quantified, and unmeasured variability in event detection may have affected phase-specific CoP estimates, particularly variables derived from short temporal windows or variables sensitive to phase duration.

The aiming phase was event-defined and variable in duration, whereas the post-discharge phase was fixed at one second and started at shot discharge. Therefore, phase comparisons involving duration-sensitive variables, especially total displacement, should be interpreted cautiously. Importantly, the observed variability in aiming-phase duration should not be interpreted solely as segmentation error, because it likely also reflects true behavioural variability in the time required to align, stabilise, and discharge the shot. In addition, because the post-discharge phase began at shot discharge, post-discharge-phase CoP variables cannot be interpreted as direct pre-shot determinants of projectile impact. The absence of direct trigger-force, firearm-motion, or recoil sensors also limits the precision with which trigger execution, shot discharge, and post-discharge recovery can be characterised. Future studies should include repeated segmentation, independent raters, direct trigger-force sensors, firearm-motion tracking, and synchronised high-speed video to improve the temporal precision and reliability of phase definition.

Peak CoP velocity variables may also be sensitive to sampling frequency, filtering, and finite-difference procedures; therefore, findings involving AP peak velocity should be interpreted as exploratory.

Future research should include larger police samples, a greater number of shots, and more ecologically valid operational scenarios involving time pressure, cognitive stress, movement, decision-making, and footwear consistent with police practice. Future studies should also consider shot-by-shot analyses, mixed-effects models, time-normalised CoP variables, direct firearm-motion tracking, trigger-force measurement, gaze behaviour analysis, electromyography, and psychophysiological measures. Such multidimensional approaches may help clarify how body composition, postural control, visual attention, trigger execution, and stress regulation interact to determine police shooting accuracy.

## 5. Conclusions

In this sample of police cadets performing a controlled short-range precision-shooting task, BIA-estimated fat mass percentage emerged as the most consistent correlate of shooting accuracy, whereas handgrip strength was not associated with performance. Although selected CoP variables were retained in exploratory models, associations between postural-control measures and shooting accuracy were generally weak and should be considered hypothesis-generating rather than confirmatory.

From a practical perspective, the findings suggest that physical conditioning and firearms training should be considered complementary components of police training programmes. However, the observed associations do not demonstrate that modifying body composition or postural-control characteristics will directly improve shooting accuracy.

The conclusions are constrained by the cross-sectional design, the limited number of shots, and the exploratory nature of the regression models. Although sex was included as a forced covariate and internal validation was performed using bootstrap resampling and leave-one-out cross-validation, the selected model-based associations may still be sample-dependent. Therefore, the practical implications should be interpreted cautiously and should not be considered evidence of causal or modifiable determinants of shooting accuracy. Future longitudinal and intervention studies involving larger police samples, more shots, and operationally realistic scenarios incorporating cognitive stress, decision-making demands, and duty footwear are needed to clarify the role of body composition and postural regulation in police precision-shooting performance.

## Figures and Tables

**Table 1:** Differences in precision-shooting performance between the 5 m and 7 m distances.

	Mean Dif.	SEM	*t*	*df*	*p*-Value	Effect Size		
						Hedges *g*	95% CI	
							Lower	Upper
7 m − 5 m score ^a^	−3.05	0.58	−5.310	56	<0.001	−0.475	−0.676	−0.275

Note: ^a^, Score from 0 to 25 (maximum); *p*, two-sided *p*-value; Hedges’ correction uses the sample standard deviation of the mean difference, plus a correction factor.

**Table 2:** Spearman correlations between morphology, handgrip strength and police shooting accuracy at 5 and 7 m.

	Descriptive Statistics						Shooting Accuracy												
					5 m							7 m							
	*n*	Mean	SD	*r*	*p*	95% CI ^a,b^		*q*	*r * ^c^	*p * ^c^	*q * ^c^	*r*	*p*	95% CI ^a,b^		*q*	*r * ^c^	*p * ^c^	*q * ^c^
						Lower	Upper							Lower	Upper				
Age (years)	57	27.67	5.11	−0.185	0.168	−0.427	0.082	0.460	−0.172	0.205	0.383	−0.180	0.180	−0.423	0.087	0.460	−0.172	0.204	0.383
Height (cm)	57	173.68	6.47	−0.090	0.507	−0.343	0.175	0.685	−0.047	0.733	0.831	−0.085	0.528	−0.339	0.180	0.685	−0.083	0.543	0.703
Body mass (kg)	57	77.81	11.89	−0.261	0.050	−0.491	0.004	0.183	−0.295	0.028	0.103	−0.279	0.036	−0.507	−0.015	0.183	−0.354	0.007	0.051
BMI (kg/m^2^)	57	25.73	3.20	−0.269	0.043	−0.498	−0.004	0.183	−0.298	0.026	0.103	−0.267	0.044	−0.497	−0.002	0.183	−0.340	0.010	0.055
BIA-estimated fat mass (%)	57	22.91	7.15	−0.302	0.022	−0.526	−0.039	0.183	−0.407	0.002	0.022	−0.345	0.009	−0.561	−0.085	0.183	−0.409	0.002	0.022
BIA-estimated muscle mass (kg)	57	56.84	9.33	−0.060	0.660	−0.315	0.204	0.691	−0.036	0.793	0.831	−0.075	0.578	−0.330	0.189	0.691	−0.111	0.416	0.610
Handgrip—right (kg)	57	46.17	9.63	−0.063	0.640	−0.319	0.201	0.691	−0.039	0.778	0.831	−0.067	0.623	−0.322	0.198	0.691	−0.171	0.209	0.383
Handgrip—left (kg)	57	44.27	9.43	−0.177	0.188	−0.420	0.090	0.460	−0.195	0.150	0.367	−0.085	0.529	−0.339	0.180	0.685	−0.217	0.109	0.343
Handgrip—right + Left (kg)	57	90.44	18.66	−0.130	0.337	−0.378	0.137	0.685	−0.121	0.376	0.591	−0.093	0.493	−0.345	0.173	0.685	−0.202	0.136	0.367
Handgrip—firing (kg)	57	44.90	8.37	−0.105	0.437	−0.356	0.161	0.685	0.095	0.485	0.667	−0.126	0.351	−0.375	0.140	0.685	0.019	0.889	0.889
Finger grip—firing (kg)	57	12.60	3.50	0.092	0.497	−0.173	0.345	0.685	−0.056	0.684	0.831	0.037	0.785	−0.226	0.295	0.785	−0.153	0.259	0.438

Note: ^a^, the estimate is based on Fisher’s r-to-z transformation; ^b^, the standard error estimate is based on the formula proposed by Bonett and Wright [20]; ^c^, partial correlation controlling for sex; *q*, Benjamini–Hochberg false-discovery-rate-adjusted *p*-value for the bivariate physical-attribute correlations; *q* ^c^, Benjamini–Hochberg false-discovery-rate-adjusted *p*-value for the sex-adjusted partial correlations.

**Table 3:** Differences in CoP variables between the aiming and post-discharge phases in police precision shooting at 5 and 7 m.

	Shooting Phases				Mean Dif.	SEM	*t*	*df*	*p*	*q*	Effect Size		
Post-Discharge Versus Aiming	Aiming		Post-Discharge								Hedges *g*	95% CI	
	Mean	SD	Mean	SD								Lower	Upper
**Distance: 5 m**													
ML amplitude (mm)	30.23	28.82	23.87	21.08	−6.36	1.66	−3.821	56	<0.001	≤0.002	−0.201	−0.312	−0.089
AP amplitude (mm)	38.99	13.51	34.78	13.22	−4.21	0.57	−7.414	56	<0.001	≤0.002	−0.310	−0.412	−0.208
ML mean velocity (mm/s)	14.80	11.92	19.94	16.95	5.14	1.17	4.388	56	<0.001	≤0.002	0.294	0.149	0.439
AP mean velocity (mm/s)	13.60	5.81	23.40	14.36	9.80	1.43	6.840	56	<0.001	≤0.002	0.648	0.422	0.874
ML peak velocity (mm/s)	219.77	225.41	158.26	135.26	−61.51	18.94	−3.247	56	0.002	0.004	−0.270	−0.444	−0.096
AP peak velocity (mm/s)	209.94	93.19	206.58	90.42	−3.36	3.47	−0.968	56	0.337	0.400	−0.036	−0.111	0.039
Total displacement (mm)	250.03	155.80	136.82	80.39	−113.21	12.62	−8.972	56	<0.001	≤0.002	−0.610	−0.788	−0.431
Ellipse area of 95% (mm^2^)	204.48	194.24	225.16	275.31	20.69	19.94	1.038	56	0.304	0.373	0.074	−0.070	0.219
Axis a (mm)	25.31	10.21	24.63	12.72	−0.68	0.94	−0.727	56	0.470	0.541	−0.055	−0.208	0.097
Axis b (mm)	8.51	4.01	8.48	5.04	−0.02	0.27	−0.089	56	0.929	0.946	−0.005	−0.108	0.099
ML RMS (mm)	5.36	3.97	5.50	4.20	0.14	0.21	0.664	56	0.509	0.553	0.033	−0.066	0.131
AP RMS (mm)	9.15	2.80	8.71	3.85	−0.44	0.35	−1.255	56	0.215	0.282	−0.121	−0.315	0.073
ML/AP amplitude	0.76	0.58	0.67	0.55	−0.09	0.03	−3.320	56	0.002	0.004	−0.149	−0.243	−0.055
ML/AP velocity	1.04	0.33	0.84	0.27	−0.19	0.03	−7.173	56	<0.001	≤0.002	−0.611	−0.817	−0.405
ML velocity/amplitude (1/s)	1.08	0.32	2.15	1.02	1.07	0.13	8.535	56	<0.001	≤0.002	1.257	0.878	1.636
AP velocity/amplitude (1/s)	0.66	0.23	1.12	0.63	0.46	0.07	6.357	56	<0.001	≤0.002	0.815	0.515	1.114
ML variance (mm^2^)	58.39	123.57	72.23	161.40	13.84	9.15	1.512	56	0.136	0.191	0.081	−0.027	0.189
AP variance (mm^2^)	96.49	62.65	103.49	109.39	7.00	10.34	0.677	56	0.501	0.553	0.067	−0.131	0.265
ML/AP covariance (mm^2^)	14.16	45.49	1.77	59.18	−12.40	3.07	−4.044	56	<0.001	≤0.002	−0.190	−0.291	−0.089
**Distance: 7 m**													
ML amplitude (mm)	29.42	27.17	21.79	20.15	−7.63	1.48	−5.157	56	<0.001	≤0.002	−0.250	−0.359	−0.142
AP amplitude (mm)	39.19	15.08	34.47	13.59	−4.73	0.60	−7.852	56	<0.001	≤0.002	−0.308	−0.405	−0.210
ML mean velocity (mm/s)	14.76	12.20	18.61	16.44	3.85	0.76	5.094	56	<0.001	≤0.002	0.180	0.101	0.258
AP mean velocity (mm/s)	13.80	6.55	22.55	14.13	8.75	1.27	6.880	56	<0.001	≤0.002	0.551	0.360	0.742
ML peak velocity (mm/s)	209.22	197.87	143.33	137.25	−65.89	12.02	−5.483	56	<0.001	≤0.002	−0.293	−0.414	−0.173
AP peak velocity (mm/s)	214.24	104.29	209.63	100.65	−4.60	2.16	−2.127	56	0.038	0.056	−0.043	−0.085	−0.002
Total displacement (mm)	252.01	160.41	136.05	104.31	−115.95	10.81	−10.729	56	<0.001	≤0.002	−0.642	−0.813	−0.471
Ellipse area of 95% (mm^2^)	205.76	230.28	204.79	299.32	−0.97	14.15	−0.069	56	0.946	0.946	−0.003	−0.084	0.079
Axis a (mm)	25.69	11.11	24.06	12.94	−1.63	0.70	−2.326	56	0.024	0.038	−0.126	−0.237	−0.015
Axis b (mm)	8.14	3.84	7.58	4.03	−0.56	0.26	−2.190	56	0.033	0.050	−0.140	−0.270	−0.009
ML RMS (mm)	5.39	4.32	5.19	4.64	−0.20	0.17	−1.144	56	0.258	0.327	−0.042	−0.116	0.032
AP RMS (mm)	9.10	2.93	8.33	3.66	−0.77	0.27	−2.809	56	0.007	0.012	−0.216	−0.375	−0.057
ML/AP amplitude	0.74	0.44	0.61	0.34	−0.13	0.03	−4.093	56	<0.001	≤0.002	−0.301	−0.458	−0.143
ML/AP velocity	1.03	0.28	0.83	0.24	−0.20	0.02	−9.762	56	<0.001	≤0.002	−0.742	−0.949	−0.535
ML velocity/amplitude (1/s)	1.10	0.27	2.21	0.94	1.11	0.11	9.937	56	<0.001	≤0.002	1.317	0.952	1.681
AP velocity/amplitude (1/s)	0.65	0.17	1.06	0.46	0.41	0.06	7.365	56	<0.001	≤0.002	1.050	0.702	1.398
ML variance (mm^2^)	79.76	217.27	92.35	277.94	12.59	8.95	1.407	56	0.165	0.224	0.022	−0.010	0.054
AP variance (mm^2^)	95.38	64.94	93.15	93.30	−2.23	7.51	−0.297	56	0.767	0.810	−0.024	−0.190	0.141
ML/AP covariance (mm^2^)	15.80	45.06	8.99	48.87	−6.81	2.86	−2.376	56	0.021	0.035	−0.141	−0.263	−0.019

Note: *p*, two-sided *p*-value; Hedges’ correction uses the sample standard deviation of the mean difference, plus a correction factor; *q*, Benjamini–Hochberg false-discovery-rate-adjusted *p*-value calculated across all 38 aiming-versus-post-discharge phase-comparison tests (statistical decisions were based on unrounded *q*-values). For *p*-values reported as <0.001, *p* = 0.001 was used for a conservative upper-bound FDR estimate.

**Table 4:** Spearman correlations between CoP variables and police shooting accuracy at 5 and 7 m, considering the aiming and post-discharge phases.

	Shooting Accuracy									
	5 m					7 m				
	*r*	*p*	95% CI ^a,b^		*q*	*r*	*p*	95% CI ^a,b^		*q*
			Lower	Upper				Lower	Upper	
**Aiming Phase**										
ML amplitude (mm)	0.224	0.095	−0.043	0.460	0.624	−0.013	0.926	−0.272	0.249	0.984
AP amplitude (mm)	0.187	0.163	−0.079	0.429	0.624	0.128	0.341	−0.138	0.377	0.646
ML mean velocity (mm/s)	0.191	0.154	−0.075	0.433	0.624	0.095	0.482	−0.170	0.347	0.733
AP mean velocity (mm/s)	0.166	0.216	−0.100	0.411	0.624	0.058	0.667	−0.206	0.314	0.819
ML peak velocity (mm/s)	0.224	0.095	−0.043	0.460	0.624	0.135	0.317	−0.131	0.383	0.624
AP peak velocity (mm/s)	0.270	0.042	0.005	0.499	0.624	0.233	0.081	−0.032	0.469	0.624
Total displacement (mm)	0.179	0.184	−0.088	0.421	0.624	0.061	0.653	−0.203	0.317	0.819
Ellipse area of 95% (mm^2^)	0.200	0.135	−0.066	0.440	0.624	−0.054	0.692	−0.310	0.210	0.835
Axis a (mm)	0.164	0.224	−0.103	0.408	0.624	0.036	0.789	−0.227	0.294	0.902
Axis b (mm)	0.184	0.171	−0.083	0.426	0.624	−0.069	0.610	−0.324	0.195	0.813
ML RMS (mm)	0.139	0.303	−0.128	0.387	0.624	−0.074	0.585	−0.328	0.191	0.794
AP RMS (mm)	0.216	0.106	−0.050	0.454	0.624	0.121	0.368	−0.145	0.371	0.650
ML/AP amplitude	0.083	0.540	−0.182	0.337	0.760	−0.206	0.125	−0.445	0.061	0.624
ML/AP velocity	0.039	0.775	−0.224	0.296	0.902	0.003	0.985	−0.258	0.263	0.985
ML velocity/amplitude (1/s)	−0.141	0.294	−0.389	0.125	0.624	0.005	0.971	−0.256	0.265	0.985
AP velocity/amplitude (1/s)	0.020	0.882	−0.242	0.279	0.958	−0.111	0.411	−0.362	0.155	0.651
ML variance (mm^2^)	0.115	0.394	−0.151	0.365	0.651	−0.155	0.249	−0.401	0.112	0.624
AP variance (mm^2^)	0.217	0.104	−0.049	0.455	0.624	0.111	0.410	−0.155	0.362	0.651
ML/AP covariance (mm^2^)	0.027	0.841	−0.235	0.286	0.940	0.058	0.668	−0.206	0.314	0.819
**Post-discharge Phase**										
ML amplitude (mm)	0.153	0.256	−0.114	0.399	0.624	0.021	0.876	−0.241	0.280	0.958
AP amplitude (mm)	0.179	0.182	−0.088	0.422	0.624	0.138	0.306	−0.128	0.386	0.624
ML mean velocity (mm/s)	0.046	0.733	−0.217	0.303	0.870	0.232	0.083	−0.034	0.467	0.624
AP mean velocity (mm/s)	0.125	0.355	−0.141	0.374	0.646	0.249	0.061	−0.016	0.482	0.624
ML peak velocity (mm/s)	0.067	0.621	−0.197	0.322	0.814	0.171	0.205	−0.096	0.414	0.624
AP peak velocity (mm/s)	0.269	0.043	0.004	0.498	0.624	0.257	0.054	−0.008	0.488	0.624
Total displacement (mm)	0.143	0.288	−0.123	0.390	0.624	0.005	0.968	−0.255	0.266	0.985
Ellipse area of 95% (mm^2^)	0.170	0.206	−0.097	0.414	0.624	0.062	0.648	−0.202	0.317	0.819
Axis a (mm)	0.116	0.388	−0.150	0.367	0.651	0.087	0.520	−0.178	0.340	0.746
Axis b (mm)	0.187	0.165	−0.080	0.428	0.624	0.088	0.515	−0.177	0.341	0.746
ML RMS (mm)	0.154	0.252	−0.112	0.400	0.624	−0.012	0.932	−0.271	0.250	0.984
AP RMS (mm)	0.179	0.182	−0.087	0.422	0.624	0.192	0.152	−0.074	0.433	0.624
ML/AP amplitude	0.003	0.984	−0.258	0.263	0.985	−0.097	0.471	−0.350	0.168	0.731
ML/AP velocity	−0.093	0.493	−0.345	0.173	0.735	−0.146	0.279	−0.393	0.121	0.624
ML velocity/amplitude (1/s)	−0.078	0.564	−0.332	0.187	0.779	0.244	0.068	−0.022	0.477	0.624
AP velocity/amplitude (1/s)	−0.112	0.407	−0.363	0.154	0.651	0.168	0.212	−0.099	0.412	0.624
ML variance (mm^2^)	0.134	0.320	−0.132	0.382	0.624	−0.035	0.795	−0.293	0.228	0.902
AP variance (mm^2^)	0.193	0.151	−0.074	0.433	0.624	0.152	0.259	−0.115	0.398	0.624
ML/AP covariance (mm^2^)	−0.140	0.298	−0.388	0.126	0.624	−0.124	0.357	−0.374	0.142	0.646

Note: ^a^, the estimate is based on Fisher’s *r*-to-*z* transformation; ^b^, the standard error estimate is based on the formula proposed by Bonett and Wright [20]. *q*, Benjamini–Hochberg false-discovery-rate-adjusted *p*-value calculated across all 76 CoP–accuracy correlations.

**Table 5:** Sex-adjusted exploratory fixed-entry regression models for police shooting accuracy at 5 and 7 m.

Model	Predictor	B	SE	Standardized β	*t*	*p*-Value	Bootstrap 95% CI	VIF
5 m	Constant	30.359	5.060	—	5.999	<0.001	20.080 to 39.049	—
	Sex	−3.818	2.167	−0.284	−1.762	0.084	−7.812 to 0.285	2.018
	BIA-estimated fat mass (%)	−0.291	0.122	−0.355	−2.378	0.021	−0.518 to −0.055	1.739
	AP peak velocity (mm/s)—aiming	0.018	0.008	0.292	2.197	0.033	0.005 to 0.034	1.380
	ML velocity/amplitude (1/s)—post-discharge	−2.232	0.712	−0.388	−3.135	0.003	−3.575 to −0.941	1.192
	ML/AP amplitude—post-discharge	−2.639	1.333	−0.247	−1.980	0.053	−6.122 to 1.690	1.212
7 m	Constant	23.025	5.242	—	4.392	<0.001	13.562 to 32.228	—
	Sex	−3.010	2.395	−0.197	−1.257	0.214	−7.609 to 1.828	1.701
	BIA-estimated fat mass (%)	−0.416	0.137	−0.447	−3.031	0.004	−0.655 to −0.183	1.511
	AP peak velocity (mm/s)—aiming	0.018	0.008	0.280	2.164	0.035	0.006 to 0.032	1.160

Note: Bootstrap confidence intervals are percentile-based 95% intervals from 5000 bootstrap samples. LOOCV, leave-one-out cross-validation; VIF, variance inflation factor. Model performance: 5 m model, *F*(5,51) = 5.371, *p* < 0.001, *R* = 0.587, *R*^2^ = 0.345, adjusted *R*^2^ = 0.281, LOOCV_*R*^2^ = 0.240, LOOCV_RMSE = 5.052, bootstrap optimism-corrected *R*^2^ = 0.237. 7-m model, *F*(3,53) = 5.468, *p* = 0.002, *R* = 0.486, *R*^2^ = 0.236, adjusted *R*^2^ = 0.193, LOOCV_*R*^2^ = 0.131, LOOCV_RMSE = 6.136, bootstrap optimism-corrected *R*^2^ = 0.156.

## Data Availability

The data supporting the findings of this study are available from the corresponding author upon reasonable request, subject to institutional, ethical, and privacy restrictions related to police training data.

## Appendix A

**Table A1:** Differences in CoP variables between 5 and 7 m police precision shooting, considering the aiming and post-discharge phases.

	Mean Dif.	SEM	*t*	*df*	*p*-Value	*q*	Effect Size		
							Hedges *g*	95% CI	
								Lower	Upper
**Aiming Phase**									
ML amplitude (mm)	−0.81	2.57	−0.316	56	0.753	0.960	−0.029	−0.210	0.153
AP amplitude (mm)	0.20	1.19	0.171	56	0.865	0.960	0.014	−0.148	0.175
ML mean velocity (mm/s)	−0.04	0.74	−0.057	56	0.955	0.960	−0.003	−0.124	0.117
AP mean velocity (mm/s)	0.20	0.42	0.480	56	0.633	0.960	0.031	−0.099	0.161
ML peak velocity (mm/s)	−10.55	14.37	−0.734	56	0.466	0.960	−0.048	−0.178	0.083
AP peak velocity (mm/s)	4.30	8.61	0.499	56	0.620	0.960	0.042	−0.128	0.213
Total displacement (mm)	1.98	8.53	0.232	56	0.817	0.960	0.012	−0.094	0.119
Ellipse area of 95% (mm^2^)	1.28	25.70	0.050	56	0.960	0.960	0.006	−0.230	0.242
Axis a (mm)	0.38	1.12	0.343	56	0.733	0.960	0.035	−0.172	0.243
Axis b (mm)	−0.36	0.41	−0.879	56	0.383	0.960	−0.091	−0.300	0.118
ML RMS (mm)	0.03	0.48	0.056	56	0.956	0.960	0.006	−0.220	0.233
AP RMS (mm)	−0.05	0.26	−0.189	56	0.851	0.960	−0.017	−0.198	0.164
ML/AP amplitude	−0.02	0.07	−0.287	56	0.775	0.960	−0.039	−0.308	0.231
ML/AP velocity	−0.01	0.04	−0.269	56	0.789	0.960	−0.032	−0.267	0.204
ML velocity/amplitude (1/s)	0.02	0.04	0.527	56	0.600	0.960	0.066	−0.185	0.317
AP velocity/amplitude (1/s)	−0.01	0.02	−0.491	56	0.625	0.960	−0.051	−0.260	0.157
ML variance (mm^2^)	21.37	28.35	0.754	56	0.454	0.960	0.116	−0.193	0.424
AP variance (mm^2^)	−1.11	5.38	−0.207	56	0.837	0.960	−0.017	−0.184	0.149
ML/AP covariance (mm^2^)	1.64	4.32	0.379	56	0.706	0.960	0.036	−0.153	0.225
**Post-discharge Phase**						0.960			
ML amplitude (mm)	−2.09	2.81	−0.742	56	0.461	0.960	−0.100	−0.370	0.170
AP amplitude (mm)	−0.31	1.10	−0.284	56	0.777	0.960	−0.023	−0.185	0.139
ML mean velocity (mm/s)	−1.33	1.52	−0.879	56	0.383	0.960	−0.079	−0.259	0.101
AP mean velocity (mm/s)	−0.85	1.03	−0.824	56	0.413	0.960	−0.059	−0.202	0.084
ML peak velocity (mm/s)	−14.93	15.99	−0.933	56	0.355	0.960	−0.108	−0.341	0.125
AP peak velocity (mm/s)	3.06	8.62	0.355	56	0.724	0.960	0.031	−0.145	0.208
Total displacement (mm)	−0.76	8.88	−0.086	56	0.932	0.960	−0.008	−0.187	0.171
Ellipse area of 95% (mm^2^)	−20.38	39.06	−0.522	56	0.604	0.960	−0.070	−0.338	0.199
Axis a (mm)	−0.57	1.45	−0.391	56	0.697	0.960	−0.044	−0.267	0.180
Axis b (mm)	−0.90	0.51	−1.764	56	0.083	0.960	−0.190	−0.408	0.029
ML RMS (mm)	−0.31	0.60	−0.510	56	0.612	0.960	−0.068	−0.337	0.201
AP RMS (mm)	−0.38	0.34	−1.116	56	0.269	0.960	−0.099	−0.278	0.080
ML/AP amplitude	−0.06	0.08	−0.789	56	0.433	0.960	−0.134	−0.474	0.207
ML/AP velocity	−0.02	0.04	−0.491	56	0.625	0.960	−0.069	−0.350	0.213
ML velocity/amplitude (1/s)	0.06	0.17	0.366	56	0.716	0.960	0.061	−0.273	0.396
AP velocity/amplitude (1/s)	−0.06	0.09	−0.728	56	0.470	0.960	−0.110	−0.413	0.193
ML variance (mm^2^)	20.12	40.01	0.503	56	0.617	0.960	0.086	−0.258	0.431
AP variance (mm^2^)	−10.34	8.77	−1.179	56	0.243	0.960	−0.098	−0.265	0.070
ML/AP covariance (mm^2^)	7.23	7.92	0.913	56	0.365	0.960	0.131	−0.157	0.418
